# THE INFLUENCE OF REVISING AN ONLINE GERONTOLOGY PROGRAM ON THE STUDENT EXPERIENCE

**DOI:** 10.1093/geroni/igz038.235

**Published:** 2019-11-08

**Authors:** Kara B Dassel, Katarina Felsted, Jacqueline Eaton

**Affiliations:** 1 University of Utah, Salt Lake City, Utah, United States; 2 University of Utah College of Nursing, Salt Lake City, Utah, United States

## Abstract

The University of Utah’s Gerontology Interdisciplinary Program (UUGIP), a fully online program recently aligned all courses to meet the 2014 Academy of Gerontology in Higher Education (AGHE) “Gerontology Competencies for Undergraduate and Graduate Education” and to meet best practices in online teaching. These efforts led to the GIP Masters of Science program being recognized in 2017 by AGHE as a Program of Merit as well as a publication in the AGHE Journal of Gerontology & Geriatrics Education (Dassel, Eaton, & Felsted, 2018). In an effort to further this work, we analyzed student evaluations in core Master of Science program courses prior to and following these program revisions, assessed by qualitative and quantitative evaluation data from two semesters immediately prior and two semesters immediately following revisions. This presentation will discuss results, implications, and future applications of this analysis.

